# Supplementary material for: A set of multi-entry identification keys to African frugivorous flies (Diptera, Tephritidae)
Source: Zookeys. 2014 Jul 24;(428):97–108. doi: 10.3897/zookeys.428.7366 (PMC4143993; doi:10.3897/zookeys.428.7366)
Supplement: Supplementary material 6 — Key to Ceratitis [file zookeys-428-097-s006.zip › SF6_ZooKeys_key to Ceratitis/key/SF6_ZooKeys_key to Ceratitis/Media/Html/Ceratitis epixantha .htm]

Ceratitis (Ceratalaspis) epixantha (Hering)


***Ceratitis*** **(*Ceratalaspis*)
*epixantha* (Hering)**

*Pardalaspis epixantha* Hering, 1941: 69.

Body
length: 4.00 mm; wing length: 4.00 mm.

Male

Head.
Antenna yellow. Third antennal segment twice as long as second segment. Arista
with short hairs over entire length. Frons with short scattered hairs which are
largely the same colour as frons; convex, in lateral view not distinctly
projecting forwards at antennal implant. Chaetotaxy normal for subgenus.

Thorax.
Postpronotum pale yellow, unspotted. Ground colour of mesonotum with dark
orange tinge. Chaetotaxy normal for subgenus. Scapular setae, one outer pale,
others missing. Two anepisternal bristles, lower one less well developed.
Scutellum white basally, otherwise yellow with three black separate markings
apically; basally without dark spots, only slight darker colouration.
Subscutellum with three separate spots at same position as apical scutellar
spots.

Legs
yellow; setation typical for subgenus, mainly pale especially on femora.
Posterior and posterodorsal rows on fore femur pale. Ventral spines pale.

Wing
bands with yellow-brown markings. Banding; marginal band continuous; cubital
band free, medial band absent; crossvein r-m well before middle of discal cell,
at basal two-fifth. Crossvein dm-cu posteriorly more inwards than anteriorly.

Abdomen.
Dark yellow. Setation and banding typical for subgenus.

Female

Unknown.
